# Desarda Non-mesh Technique Versus Lichtenstein Technique for the Treatment of Primary Inguinal Hernias: A Systematic Review and Meta-Analysis

**DOI:** 10.7759/cureus.31630

**Published:** 2022-11-18

**Authors:** Chirag Pereira, Benji Varghese

**Affiliations:** 1 General Surgery, Royal Lancaster Infirmary, Lancaster, GBR; 2 Urology, Manchester Royal Infirmary, Manchester, GBR

**Keywords:** systematic review and meta-analysis, inguinal hernia repair, rct, lichtenstein’s repair, desarda repair

## Abstract

The aim of the current systematic review was to compare the Desarda technique with the Lichtenstein technique for the repair of inguinal hernias. A systematic literature search for randomized controlled trials* (*RCTs) was conducted comparing the Desarda technique and the Lichtenstein technique using electronic databases. The primary outcome evaluated was hernia recurrence and the secondary outcomes evaluated were post-operative complications, time to return to normal activity and operative time in minutes. Five RCTs were included in this meta-analysis, which included a total of 536 patients. There were 310 patients in the Desarda group and 226 patients in the Lichtenstein group. There was no statistically significant difference in terms of hernia recurrence between the two groups (p=0.74). Post-operative complications were significantly more in the Lichtenstein group. There was a lower rate of post-operative seroma following the Desarda technique as compared to Lichtenstein repair (p=0.02). Both Desarda and Lichtenstein had low rates of recurrence following surgery and had acceptable rates of post-operative complications with lower rates noted in the Desarda group.

## Introduction and background

One of the most common operations performed by general surgeons is inguinal hernia repair. The estimated incidence of inguinal hernias is 15% in the adult population [1], and the lifetime risk of developing a hernia is nearly 30% in males and 3% in females [2].

There have been numerous surgical techniques for the repair of inguinal hernias that have been described in the literature. In 2009, the European Hernia Society (EHS) strongly recommended the use of the Lichtenstein technique (LT) for the repair of primary inguinal hernias based on the analysis of several clinical trials [3].

LT is a tension-free repair of the inguinal canal making use of a synthetic mesh to reinforce the posterior wall of the inguinal canal. Although it is one of the most commonly performed operations for inguinal hernias, the use of a prosthetic mesh has resulted in an increased incidence of foreign body sensation and chronic groin pain [4]. The Desarda technique (DT) described by its pioneer, Prof. M.P. Desarda, makes use of a sling of the external oblique of the patient to reinforce the posterior wall making it a non-mesh repair for inguinal hernias and hence reducing mesh-related complications [5].

The aim of the current systematic review is to compare non-mesh-based DT with mesh-based LT for the repair of primary inguinal hernias. The main outcome parameters were hernia recurrence on follow-up. The secondary outcomes are overall postoperative complications, operative time and duration needed to return to normal activity following surgery.

## Review

This systematic review and meta-analysis was designed and reported according to Preferred Reporting Items for Systemic Reviews and Meta-Analyses (PRISMA) [6].

Search strategy

**Figure 1:**
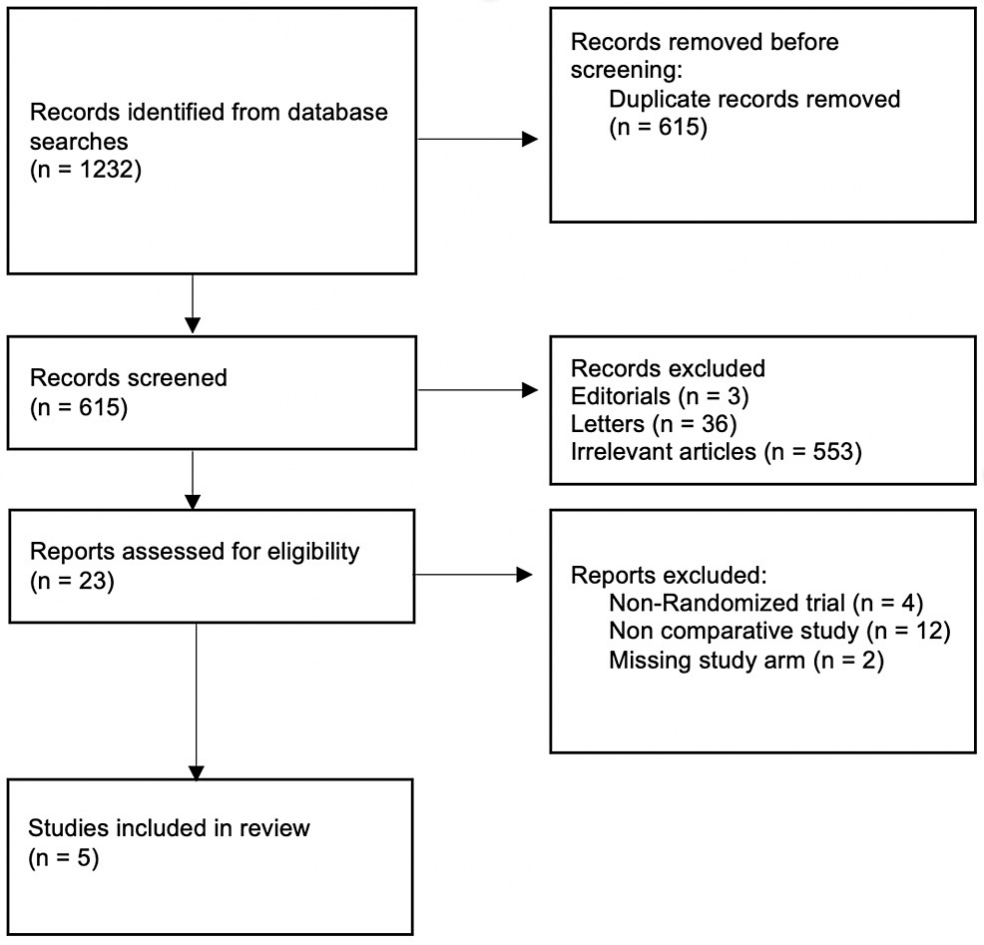
PRISMA Flow Diagram PRISMA: Preferred Reporting Items for Systemic Reviews and Meta-Analyses

A literature search for RCTs comparing Desarda repair and Lichtenstein repair was conducted by two different investigators independently. PubMed/Medline and Embase using the OVID interface were the two databases used to conduct the search. Studies were limited to English articles and human studies and there were no publication date restrictions. Keywords used for electronic searches were ‘Desarda’, ‘Lichtenstein’, and ‘hernia repair’ (Figure 1).

Study selection

Studies that were included for review were (1) all original RCTs; (2) patients undergoing Desarda repair for primary inguinal hernias and (3) a control group undergoing Lichtenstein hernia repair. Excluded studies were non-RCTs, case reports, editorials, observational studies, reviews and meta-analyses.

Data collection

The following data were collected from each study: first author and year of publication, study country of origin, population distribution, gender distribution, mean age, type of hernia and length of follow-up. The main outcome was hernia recurrence during the follow-up period while the secondary outcomes were the overall post-operative complications, time to return to normal activity and operative time in minutes. Data were collected by two authors separately and inputted into a Microsoft Excel sheet (Microsoft Corporation, Redmond, WA) to ensure conformity.

Assessment of risk of bias

**Figure 2:**
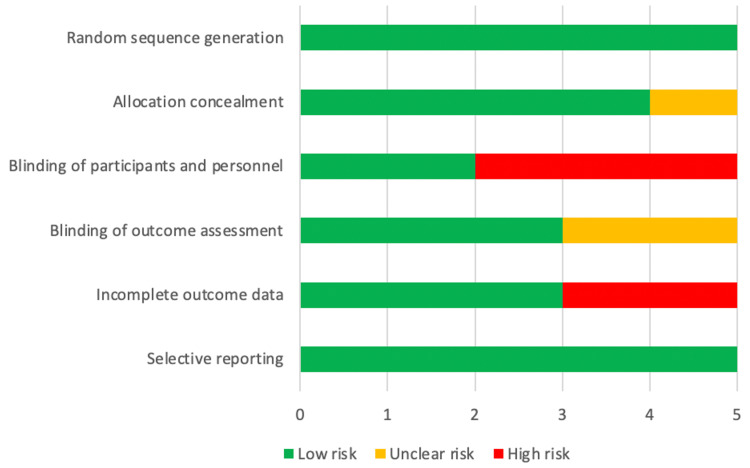
Assessment of Risk of Bias

The risk of bias was assessed based on the Cochrane risk of bias tool by two authors [7]. The following categories were classified as low, high or unclear: random sequence generation, allocation concealment, blinding of outcome assessment, blinding of participants and personnel, selective reporting and other sources of bias. Discrepancies in the interpretation of the risk of bias were resolved by mutual agreement between authors (Figure 2).

Statistical analysis

Review Manager 5.4 (The Cochrane Collaboration, 2020) was used for statistical analysis. The mean difference at a 95% confidence interval was calculated for continuous variables and the risk ratio at a 95% confidence interval was calculated for dichotomous variables. Random effect models and fixed effect models were used for analysis appropriately. The Cochrane Q test and I2 test were used to assess heterogeneity in the included studies. Zero per cent (0%) was considered no heterogeneity while >50% was considered significant heterogenicity. The chi-square test was used to assess the significance of post-operative complications. A p of <0.05 was considered to be statistically significant.

Results

Five RCTs were included in the meta-analysis with a total number of 536 patients. There were 310 patients in the Desarda group and 226 patients in the Lichtenstein group. In terms of hernia laterality, there were 333 right-sided inguinal hernias and 191 left-sided inguinal hernias. Only one study reported 12 bilateral hernias, however, only one side was operated on. Characteristics of the included studies and population characteristics are shown in Table 1 and Table 2, respectively.

**Table 1:** Characteristics of Included Studies COPD = Chronic obstructive pulmonary disease; ASA = American Society of Anaesthesiologists; TB = Tuberculosis; DM = Diabetes mellitus

Study	Country (Year of publication)	Population distribution (DT/LT)	Method of randomization	Description of population	Length of follow up
Manyilirah et al. [8]	Uganda (2012)	101 (50/51)	Computer-generated randomization and sealed envelope	Exclusion criteria: Giant inguinoscrotal hernias, obstructive uropathy, chronic obstructive pulmonary disease (COPD), impaired mental state	2 weeks
Szopinski et al. [9]	Poland (2012)	208 (105/103)	Not mentioned	Exclusion criteria: Recurrent or strangulated hernias, American Society of Anaesthesiologists (ASA) scale 3, history of a forced hernia reduction with subsequent hospitalization, History of infection scar in the inguinal area	36 months
Youssef et al. [10]	Egypt (2015)	143 (71/72)	Sealed envelope	Exclusion criteria: Less than 18 years scar in inguinal region strangulated, recurrent or giant inguinoscrotal hernia, history of forced hernia reduction with subsequent hospitalization, poorly controlled Diabetes Mellitus (DM), chronic cough, those on TB treatment, severe hypertension, COPD, obstructive uropathy, ASA > 3 major psychological instability and drug abuse. Patients found to have thin, weak or divided external oblique aponeurosis (EOA) intraoperatively	24 months
Arafa et al. [11]	Egypt (2020)	80 (40/40)	Computer-generated randomization and sealed envelope	Exclusion criteria: Under the age of 18 years, a scar in the inguinal region, recurrent or giant inguinoscrotal hernia, poorly controlled DM, chronic cough and COPD, thin, weak, or divided EOA intraoperatively, lost to follow-up within 2 years	24 months
Jain et al. [12]	India (2021)	84 (44/40)	Randomization by sealed envelope	Exclusion criteria: Less than 18 years of age, strangulated, recurrent, irreducible, obstructed hernias, chronic cough/COPD, uncorrected bladder outlet obstruction local skin infection	6 months

**Table 2:** Characteristics of the Population DT = Desarda technique; LT = Lichtenstein technique

Study	Age	Gender Male: Female	Right-sided Hernia	Left-sided Hernia	Bilateral hernias
Manyilirah et al. [8]	DT 40, LT 28.5	DT 46:4, LT 42:9	DT 29, LT 34	DT 21, LT 17	DT 0, LT 0
Szopinski et al. [9]	DT 50.2 ± 17.5, LT 54.1 ± 15.3	All males	DT 59, LT 54	DT 42, LT 41	DT 4, LT 8
Youssef et al. [10]	DT 45.97 ± 10.69, LT 43.9 ± 10.27	DT 69:2, LT 69:3	DT 49, LT 56	DT 22, LT 16	DT 0, LT 0
Arafa et al. [11]	DT 32.8 ± 7.9, LT 34.65 ± 8.12	Not mentioned	DT 8, LT 13	DT 32, LT 27	DT 0, LT 0
Jain et al. [12]	DT 48.82 ± 15.71, LT 48.53 ± 14.78	All males	DT 26, LT 26	DT 18, LT 14	DT 0, LT 0

Clinical outcomes of the DT and LT groups with respect to recurrence, overall complications and operative time are shown in Table 3.

**Table 3:** Clinical Outcomes in the Included Studies DT = Desarda technique; LT = Lichtenstein technique

Study	Recurrence on follow-up	Complications	Operative time (min)
Manyilirah et al. [8]	DT 0/49, LT 0/49	DT 7/50, LT 9/51	DT 10, LT 15.9
Szopinski et al. [9]	DT 2/105, LT 2/103	DT 26/105, LT 32/103	Not Applicable
Youssef et al. [10]	DT 1/71, LT 1/72	DT 14/71, LT 16/72	DT 59.4, LT 72.3
Arafa et al. [11]	DT 2/40, LT 1/40	DT 7/40, LT 21/40	DT 57.4, LT 68.78
Jain et al. [12]	DT 0/44, LT 0/40	DT 9/44, LT 22/40	DT 31.3, LT 65.5

Hernia recurrence

**Figure 3:**
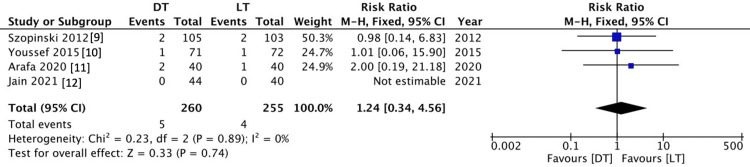
Comparison of Recurrence Between the DT and LT Groups DT = Desarda technique; LT = Lichtenstein technique

Four studies followed up on patients for six months or more to evaluate hernia recurrence. Nine patients out of 515 patients had a recurrent hernia. There was no statistically significant difference between hernia recurrence between the Desarda group and the Lichtenstein group (RR 1.24 (0.34 - 4.56), 95% CI, P = 0.74). There was a low level of heterogeneity (I2 = 0%, P = 0.89) (Figure 3).

Operative complications

**Figure 4:**
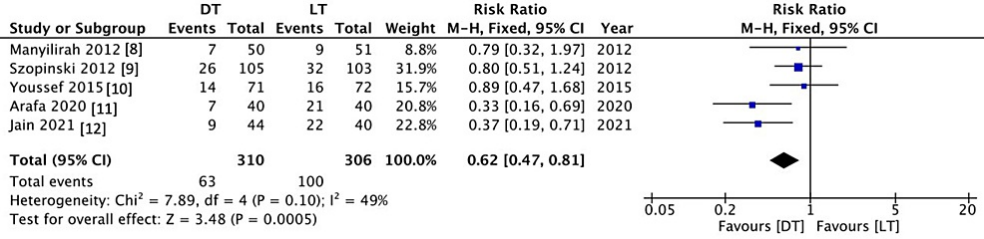
Comparison of Overall Complications Between the DT and LT Groups DT = Desarda technique; LT = Lichtenstein technique

Overall complications were reported in all included studies in this systemic review out of 613 patients. The difference in the risk of post-operative complications was significantly higher in the Lichtenstein group as compared to the Desarda group (25.5% versus 48.5%, respectively, RR 0.62 (0.47 - 0.81), 95% CI, P = 0.0005). There was moderate heterogeneity among the included studies (I2 = 49%, P = 0.10) (Figure 4).

There were 51 (16.4%) and 79 (25.8%) overall complications recorded in the DT and LT groups, respectively. The most common complication was hematoma occurring in 34 patients overall. There were 15 (4.9%) patients out of 306 that developed a post-operative seroma in the LT group and five (1.6%) out of 310 patients in the DT group (p = 0.02) (Table 4).

**Table 4:** Summary of Complications in the DT and LT Groups DT = Desarda technique; LT = Lichtenstein technique

Complications	DT (N=310)	LT (N=306)	Total	P value
Surgical site infection	3	7	10	0.19
Hematoma	14	20	34	0.27
Seroma	5	15	20	0.02
Testicular oedema	8	10	18	0.61
Hydrocele	1	0	1	
Ecchymosis	5	5	10	0.98
Scrotal oedema	12	18	30	0.24
Orchitis	3	4	7	0.69

Operative time

**Figure 5:**
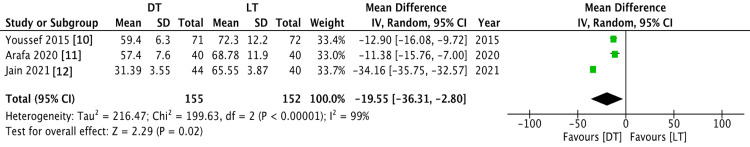
Comparison of Total Operative Time Between the DT and LT Groups DT = Desarda technique; LT = Lichtenstein technique

Total operative time was reported in four out of the five trials. Manyilirah et al. reported operative time from skin incision to the last stitch being knotted prior to closing the layers of the surgical wound and hence was also excluded from calculating the operative time. There was a statistically significant difference between the DT and LT groups with respect to operative times (MD -19.55 (-36.1-2.8), 95% CI, P = 0.02). There was a high level of heterogeneity in the included studies (I2 = 99%, P = 0.00001) (Figure 5).

Return to normal activity

**Figure 6:**
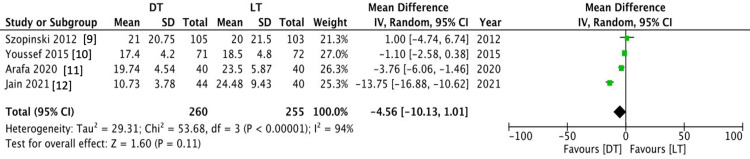
Comparison Time to Return to Normal Activity Between the DT and LT Groups DT = Desarda technique; LT = Lichtenstein technique

Return to normal daily activity was reported in four RCTs that included 515 patients. There was no statistically significant difference between the LT and DT groups (MD -4.56 (-10.13-1.01), 95% CI, P = 0.11). The level of heterogeneity was high among the included studies (I2 = 94%, P = 0.00001) (Figure 6).

Discussion

Inguinal hernia repair can be classified as either a tissue-based repair or a mesh-based repair. For many years, Bassini’s tissue-based repair was considered to be a standard approach for the repair of inguinal hernias [10]. The main limitation of this repair was that it puts surrounding tissue into tension, which resulted in a higher recurrence rate of about 8.6% [13]. Shouldice repair is also a tissue-based repair but has a much lower rate of recurrence of 1% however it is technically demanding and can have high rates of recurrence if done incorrectly [14]. Lichtenstein repair is based on the concept of tension-free repair. It makes use of a synthetic mesh to reconstruct the posterior wall of the inguinal canal and produce fibrosis so as to reduce the risk of recurrence. The downfall of using a synthetic mesh is the greater potential for post-operative complications, which include seroma, surgical site infection, impaired testicular function and chronic groin pain [15]. Desarda repair seems to overcome these complications as it makes use of the patient's own external oblique to reconstruct the inguinal canal.

The present systematic review included five RCTs which compared DT with LT. There were no significant differences in the baseline characteristics of the patients included in the various studies included in this review. The majority of the patients included were male. During data analysis to look for recurrence, one of the RCTs was excluded when looking at the recurrence rate as patients were not followed up for six months [8]. There was no statistically significant difference in the recurrence of inguinal hernias in either of the two groups.

In terms of post-operative complications, post-operative hematomas were the most common post-operative complication encountered and were more common in the LT group but were not statistically significant. A post-operative seroma was more commonly seen in the LT group as compared to the DT group and this was statistically significant. Surgical site infection is rare to occur following elective inguinal hernia surgery and can be very difficult to manage if it does occur. Surgical site infection was higher in the LT group, as this invariably involves the use of foreign material to reconstruct the inguinal canal. The other complications documented in the included RCTs were testicular oedema, ecchymosis, hydrocele, scrotal oedema and orchitis. There was no statistically significant difference between the LT and DT groups and this is comparable to a similar systematic review by Emile et al. [15].

When analyzing the operative time, we had a exclude one RCT to maintain conformity in the analysis [9]. Our analysis showed that DT repair required less time to perform as compared to LT repair and was statistically significant. This could potentially be due to more time required to place a mesh in its appropriate position as the main aim is to repair the inguinal canal as well as reduce the chances of recurrence. Mohamedamed et al. in their review had similar findings but failed to show a statistical significance [16]. Four RCTs reported a return to normal activity. There was no statistically significant difference between patients in relation to the return to normal activity.

The limitations of this meta-analysis are that the follow-up period varied among the RCTs included. There was no uniformity in post-operative pain assessment among the included studies, hence this could not be assessed in the meta-analysis. There is no mention of the type of mesh used in the studies, which may affect the primary and secondary outcomes of the systematic review.

## Conclusions

The findings of the present systematic review are comparable to those done in the past and show that LT and DT are comparable in terms of recurrence rates. Looking at other secondary outcomes, post-operative complications especially seroma after surgery was more in the LT group as compared to the DT group. We recommend prospective, multicenter, well-structured RCTs with longer follow-up periods in order to further evaluate DT.

## References

[1] Awad SS, Fagan SP (2004). Current approaches to inguinal hernia repair. Am J Surg.

[2] Primatesta P, Goldacre MJ (1996). Inguinal hernia repair: incidence of elective and emergency surgery, readmission and mortality. Int J Epidemiol.

[3] Simons MP, Aufenacker T, Bay-Nielsen M (2009). European Hernia Society guidelines on the treatment of inguinal hernia in adult patients. Hernia.

[4] Just E, Botet X, Martínez S, Escolà D, Moreno I, Duque E (2010). Reduction of the complication rate in Liechtenstein hernia repair. Int J Surg.

[5] Gedam BS, Bansod PY, Kale VB, Shah Y, Akhtar M (2017). A comparative study of Desarda's technique with Lichtenstein mesh repair in treatment of inguinal hernia: a prospective cohort study. Int J Surg.

[6] (2022). Cochrane Handbook for Systematic Reviews of Interventions. https://training.cochrane.org/handbook/current.

[7] Higgins JP, Altman DG, Gøtzsche PC (2011). The Cochrane Collaboration's tool for assessing risk of bias in randomised trials. BMJ.

[8] Manyilirah W, Kijjambu S, Upoki A, Kiryabwire J (2012). Comparison of non-mesh (Desarda) and mesh (Lichtenstein) methods for inguinal hernia repair among black African patients: a short-term double-blind RCT. Hernia.

[9] Szopinski J, Dabrowiecki S, Pierscinski S, Jackowski M, Jaworski M, Szuflet Z (2012). Desarda versus Lichtenstein technique for primary inguinal hernia treatment: 3-year results of a randomized clinical trial. World J Surg.

[10] Youssef T, El-Alfy K, Farid M (2015). Randomized clinical trial of Desarda versus Lichtenstein repair for treatment of primary inguinal hernia. Int J Surg.

[11] Ge H, Liang C, Xu Y, Ren S, Wu J (2018). Desarda versus Lichtenstein technique for the treatment of primary inguinal hernia: A systematic review. Int J Surg.

[12] Jain SK, Bhatia S, Hameed T, Khan R, Dua A (2021). A randomised controlled trial of Lichtenstein repair with Desarda repair in the management of inguinal hernias. Ann Med Surg (Lond).

[13] Hay JM, Boudet MJ, Fingerhut A (1995). Shouldice inguinal hernia repair in the male adult: the gold standard? A multicenter controlled trial in 1578 patients. Ann Surg.

[14] Junge K, Rosch R, Klinge U (2006). Risk factors related to recurrence in inguinal hernia repair: a retrospective analysis. Hernia.

[15] Emile SH, Elfeki H (2018). Desarda's technique versus Lichtenstein technique for the treatment of primary inguinal hernia: a systematic review and meta-analysis of randomized controlled trials. Hernia.

[16] Mohamedahmed AY, Ahmad H, Abdelmabod AA, Sillah AK (2020). Non-mesh Desarda technique versus standard mesh-based Lichtenstein technique for inguinal hernia repair: a systematic review and meta-analysis. World J Surg.

